# Evaluation of the Unintended Effects of Herbicide-Resistant Soybean Seeds via TMT Quantitative Proteomics and Flavonoid-Targeted Metabolomics

**DOI:** 10.3390/ijms27020734

**Published:** 2026-01-11

**Authors:** Zhanchao Wang, Ruizhe Wang, Mei Dong, Guihua Hu, Chaohua Miao, Yusong Wan, Weixiao Liu, Wujun Jin

**Affiliations:** 1 Biotechnology Research Institute, Chinese Academy of Agricultural Sciences, Beijing 100081, China; w1551725922@126.com (Z.W.); dmei2010@126.com (M.D.); huguihua@caas.cn (G.H.); miaochaohua@caas.cn (C.M.); wanyusong@caas.cn (Y.W.); 2 National Nanfan Research Institute, Chinese Academy of Agricultural Sciences, Sanya 572024, China

**Keywords:** unintended effect evaluation, soybean seeds, TMT quantitative proteomics, DEP, flavonoid-targeted metabolomics, herbicide-resistant soybean varieties, natural genotypic soybean varieties

## Abstract

Tandem mass tag (TMT) quantitative proteomic and flavonoid-targeted metabolomic analyses were applied to evaluate the unintended effects of five herbicide-resistant soybean varieties, in addition to three natural genotypic soybean varieties. A total of 65, 29, 56, 38, and 26 differentially expressed proteins (DEPs) were identified in ZLD6010, FD3003, JY2812, ZLD8001, and ZLD2426, respectively, compared with ZH13. Twenty-four and 16 DEPs were identified in ZLD2426 compared with JD12 and KS1, respectively. Kyoto Encyclopedia of Genes and Genomes (KEGG) pathway enrichment analysis revealed that most of the DEPs were involved in ribosome, protein processing in the endoplasmic reticulum, and tropane, piperidine, and pyridine alkaloid biosynthesis. Proteomic analysis of the studied soybean seeds revealed no significant changes in herbicide-resistant soybean varieties compared with natural genotypic soybean varieties. Flavonoid-targeted metabolomics analysis detected and quantified 12 flavonoids. Daidzein, genistein/apigenin, taxifolin, and luteolin contents in the herbicide-resistant soybean variety seeds were significantly greater than those in the natural genotypic soybean variety seeds. Their contents in the seeds of three natural genotypic soybean varieties also significantly differed according to pairwise reciprocal comparisons. The introduction of 3-phosphoshikimate 1-carboxyvinyltransferase (EPSPS) might affect flavonoid accumulation of herbicide-resistant soybean varieties. The genetic background of soybean also influences its flavonoid metabolomic profile.

## 1. Introduction

Weeds compete with crops for water, nutrients, space, light, and other growth resources and may become hosts of insect pests, thus severely restricting agricultural production and leading to crop yield reduction [1]. Genetically modified (GM) crops are modified with the exogenous genes *epsps*, *pat*, *tfdA*, *aad,* and *dmo*, which synthesize proteins that confer herbicide resistance to crops [1,2,3]. The bacterial *epsps* gene, which encodes 5-enolpyruvylshikimate-3-phosphate synthase, confers glyphosate tolerance in crops. Similarly, the *pat* gene encodes glyphosate acetyltransferase, which also imparts glyphosate resistance. The *tfda* gene encodes α-ketoglutarate-dependent dioxygenase (TfdA protein), enabling herbicide tolerance through the degradation of 2,4-D herbicides. The *aad* gene encodes arylalkanoate dioxygenase (AAD enzyme), which can directly degrade herbicide compounds such as phenoxy auxins and arylphenoxypropionate esters that enter the plant. Additionally, the *dmo* gene encodes dicamba monooxygenase, thereby conferring tolerance to dicamba herbicides in genetically modified crops. However, the insertion of exogenous genes may lead to deletion, insertion, or rearrangement of genes, thereby affecting some biological pathways or the formation of new allergens or toxins [4,5]. Thus, the unintended effects of GM crops must be carefully and comprehensively evaluated [6,7].

Omics techniques provide a comprehensive evaluation of the transcripts, proteins, and metabolites of GM crops [8,9,10,11,12]. In particular, proteomics and metabonomics analyses have been widely used to directly reveal the unintended effects of GM crops at the protein and metabolite levels, which are directly related to crop phenotypes [13,14,15,16,17,18,19,20]. TMT quantitative proteomics enables simultaneous comparison of protein content across multiple samples. Targeted metabolomics allows specific detection and accurate quantification of metabolite groups using standards. Flavonoids, plant secondary metabolites derived from 2-phenylchromogenone, including the isomers of flavonoids and their hydrogenation and reduction products, are important metabolites that are abundant in soybean seeds.

Transgenic herbicide-tolerant crops grown worldwide mainly include soybean, corn, cotton, and rape, among which transgenic corn and soybean have the largest planting areas [2,3]. Soybean is an economically important crop. GM soybeans provide a large amount of food for human consumption and animal feed. Their planting area now constitutes more than 50% of that covered by all GM crops. Soybean seeds, which are storage and edible tissues that contain abundant protein and flavonoids, are particularly suitable for studying the unintended effects. In this study, TMT quantitative proteomic and flavonoid-targeted metabolomic analyses were applied to evaluate the unintended changes in the herbicide-resistant soybean seeds of the varieties ZLD6010, ZLD8001, and ZLD2426 (which carry the *gat* and *g2-epsps* genes), FD3003 (which carries the *pat* and *cp4-epsps* genes), and JY2812 (which carries the *g10-epsps* genes). These selected GM soybean varieties show excellent herbicide resistance and have great application prospects. Based on these omics data, the changes in protein and flavonoid profiles will be elucidated. This approach is conducive to promoting the development of these GM soybean varieties.

## 2. Results

### 2.1. Protein Profile of Soybean Seeds

**Figure 1:**
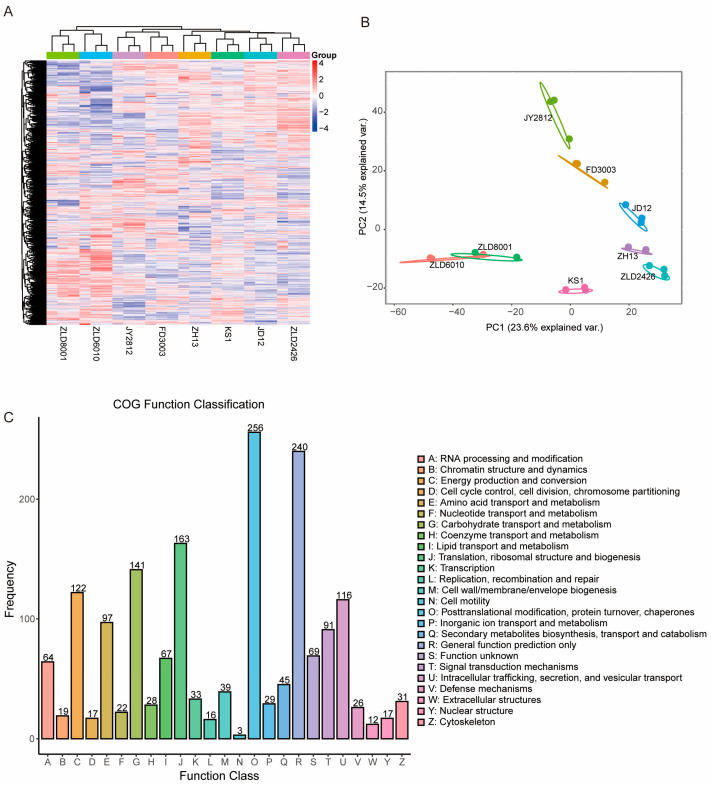
Proteomic profile of the studied soybean variety seeds. (**A**) Cluster map comparing the protein expression patterns in the seeds of eight studied soybean varieties. Red indicates relatively high expression, blue indicates relatively low expression, and white indicates the same expression levels in the two lines. All MS data were normalized and then subjected to cluster analysis. (**B**) Principal component (PC) analysis of the protein levels in the seeds of eight studied soybean varieties. Score plot of the first two PCs with the explained variance. (**C**) COG functional classes of all the identified proteins. The letters under the X-axis represent the COG categories listed on the right of the column, and the Y-axis represents the number of proteins.

The identities of the studied soybean lines were first confirmed by gene-specific PCR. Specific PCR fragments were only obtained from herbicide-tolerant soybean lines, as expected (Figure S1). A total of 4371 proteins were successfully detected in the soybean seeds. Cluster analysis of the identified proteins revealed that ZLD8001 and ZLD6010, and the other six soybean varieties, were divided into two groups. Among the other six soybean varieties, ZLD2426 and JD12 had the highest similarity. JD12 and KS1 were more similar to each other than were JD12 and ZH13. Compared with those of ZLD6010 and ZLD8001, the three natural genotypic soybean varieties had greater similarity to FD3003 and JY2812 (Figure 1A). PCA showed a high degree of similarity in the samples from the biological replicates, and revealed that PC1 (23.60%) and PC2 (14.50%) were the two main components (Figure 1B).

The identified proteins were classified into 25 clusters of orthologous group (COG) categories (Figure 1C). Among these categories, posttranslational modification, protein turnover, chaperones, general function prediction only, and translation, ribosomal structure and biogenesis represented three of the largest groups associated with general protein function, protein biogenesis, and modification in soybean seeds. These groups were followed by carbohydrate transport and metabolism, energy production and conversion, intracellular trafficking, secretion, and vesicular transport. Finally, low-abundance categories, such as cell motility, extracellular structures, and replication, recombination, and repair, accounted for a low proportion (<1%) of the identified proteins, which was consistent with the functions of these proteins in soybean seeds.

### 2.2. DEP Detection in Soybean Seeds

The number and regulatory state of the DEPs in the different comparison groups are summarized in Table 1. There were 29, 18, and 8 upregulated proteins and 36, 20, and 18 downregulated proteins, corresponding to a total of 65, 38, and 26 DEPs identified in the ZLD6010/ZH13, ZLD8001/ZH13, and ZLD2426/ZH13 comparison groups, respectively (Tables S1–S3). A total of 29 and 56 DEPs were identified via comparison of FD3003 and JY2812 with ZH13, with 15 and 21 upregulated proteins and 14 and 35 downregulated proteins, respectively (Tables S4 and S5). A total of 24 and 16 DEPs were identified in the ZLD2426/JD12 and ZLD2426/KS1 comparison groups, including 9 and 6 upregulated proteins and 15 and 10 downregulated proteins, respectively (Tables S6 and S7). Additionally, 27, 20, and 25 DEPs were identified in the JD12/ZH13, ZH13/KS1, and JD12/KS1 comparison groups, respectively (Tables S8–S10).

### 2.3. KEGG Pathway Enrichment Analysis of the Identified DEPs

**Figure 2:**
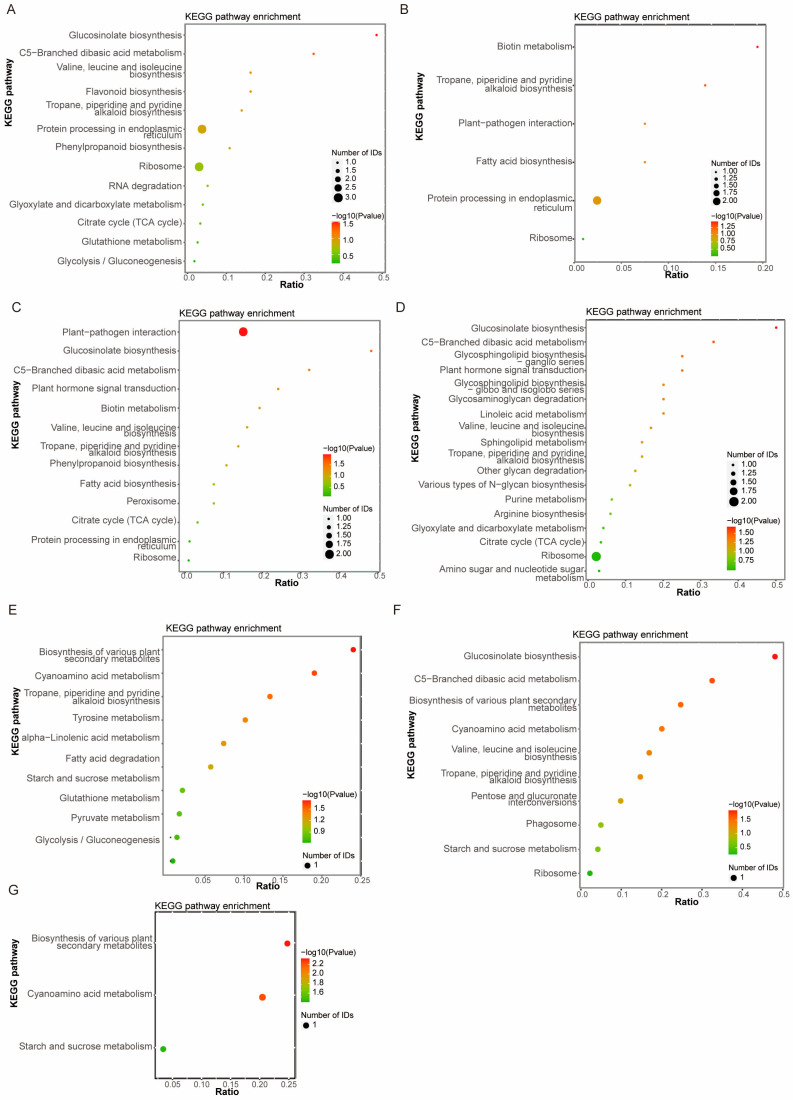
KEGG pathway enrichment analysis of DEPs in ZLD6010/ZH13 (**A**), FD3003/ZH13 (**B**), JY2812/ZH13 (**C**), ZLD8001/ZH13 (**D**), ZLD2426/JD12 (**E**), ZLD2426/ZH13 (**F**) and ZLD2426/KS1 (**G**).

KEGG pathway enrichment analysis revealed that the DEPs identified in the ZLD6010/ZH13 comparison group were involved mainly in ribosome, protein processing in endoplasmic reticulum and flavonoid biosynthesis (Figure 2A); the DEPs identified in the FD3003/ZH13 comparison group were involved mainly in protein processing in endoplasmic reticulum (Figure 2B); the DEPs identified in the JY2812/ZH13 comparison group were involved mainly in plant–pathogen interactions (Figure 2C); the DEPs identified in the ZLD8001/ZH13 comparison group were involved mainly in ribosome (Figure 2D); followed by other pathways, such as tropane, piperidine and pyridine alkaloid biosynthesis, C5-branched dibasic acid metabolism, citrate cycle (TCA cycle), glucosinolate biosynthesis, and valine, leucine and isoleucine biosynthesis. The DEPs obtained from the ZLD2426/(3 natural genotypic soybean varieties, JD12, ZH13, and KS1) comparison groups were involved mainly in the biosynthesis of various plant secondary metabolites, cyanoamino acid metabolism, and starch and sucrose metabolism (Figure 2E–G). The DEPs from pairwise comparisons of three natural genotypic soybean varieties were mainly involved in biosynthesis of various plant secondary metabolites, cyanoamino acid metabolism, glutathione metabolism, and starch and sucrose metabolism (Figure S2).

### 2.4. Identification of Co-DEPs in Soybean Seeds

**Figure 3:**
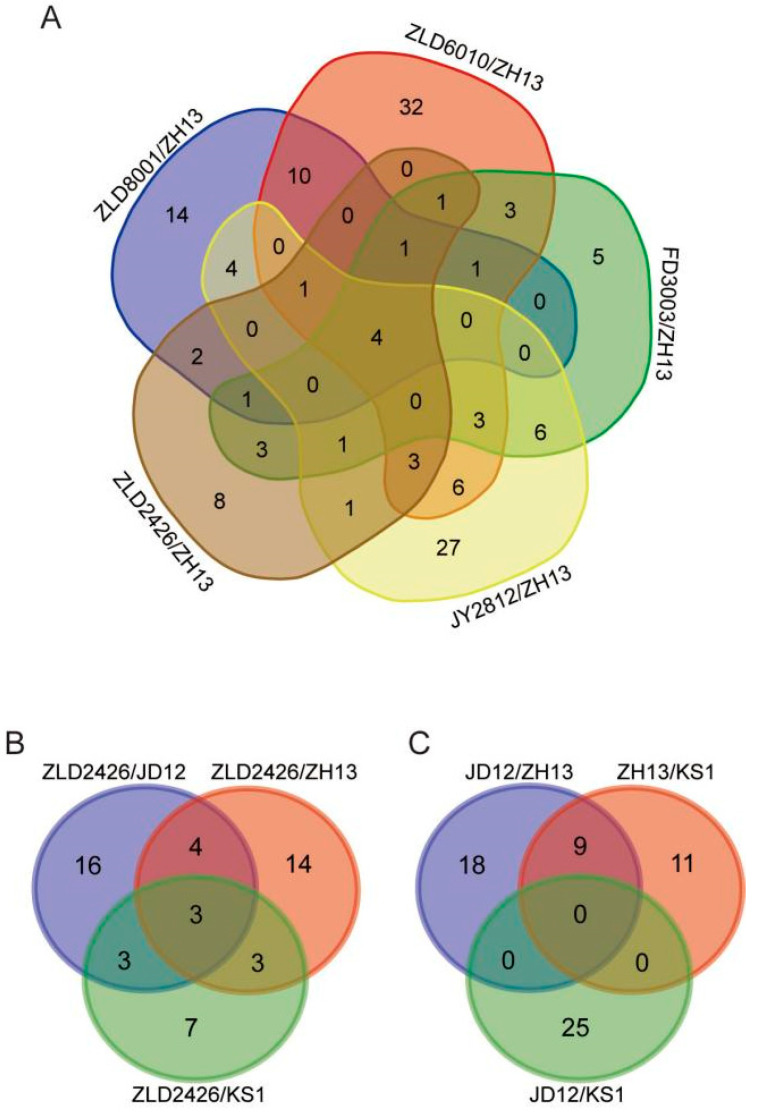
Venn diagram showing the number of overlapping DEPs identified from five herbicide-resistant soybean varieties compared with ZH13 (**A**), ZLD2426 compared with three natural genotypic soybean varieties (**B**), and pairwise comparisons between three natural genotypic soybean varieties (**C**).

Four co-DEPs were identified across all five herbicide-resistant varieties compared to ZH13 (Figure 3A). Acyl-[acyl-carrier-protein] desaturase and 40S ribosomal protein S21 were consistently downregulated. A0A0R4J4R5 showed consistent upregulation. The translation elongation factor EF1B showed variable regulation, being upregulated in FD3003/ZH13 but downregulated in the other four comparisons (Table 2). The expression levels of the translation elongation factor EF1B, also determined by RT-qPCR, were largely consistent with the omics profiling results. Compared to ZH13, the expression level was significantly downregulated in ZLD soybean lines, significantly upregulated in FD3003, and showed no significant difference in JY2812 (Figure S3). Three co-DEPs, 40S ribosomal protein S4, phosphoglucomutase, and hydrophobic seed protein, were identified in the three comparison groups (ZLD2426/natural genotypic soybean varieties JD12, ZH13, and KS1, respectively) (Figure 3B), and all were downregulated (Table 3). There were no co-DEPs in the reciprocal comparison groups of the three natural genotypic soybean varieties (Figure 3C).

### 2.5. Exogenous Protein Detection by ELISA and Proteomic Analysis of Seeds of GM Soybean Varieties

The ELISA data showed that the contents of the exogenous proteins, G2 EPSPS and GAT, were 79.25, 150.84, and 93.66 ng/g, and 14.78, 34.71, and 71.43 ng/g in the ZLD6010, ZLD8001, and ZLD2426 soybean seeds, respectively. The contents of CP4 EPSPS and PAT were 186.05 and 4.36 μg/g, respectively, in FD3003 seeds. The content of G10 EPSPS was 19.80 μg/g in JY2812 seeds. Proteomic data revealed that G10 EPSPS was a DEP in JY2812/ZH13, and CP4 EPSPS, but not PAT, was identified as a DEP in FD3003/ZH13. Neither GAT nor G2 EPSPS were identified as DEPs in ZLD soybean seeds (Table 4).

### 2.6. Flavonoid Detection in Soybean Seeds by Targeted Metabolomics Analysis

**Figure 4:**
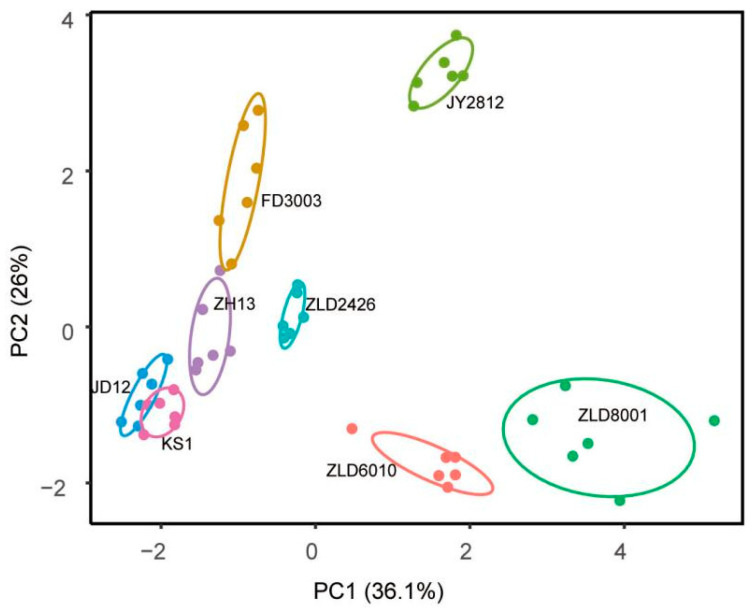
Principal component (PC) analysis of flavonoid levels in seeds of eight studied soybean varieties. Score plot of the first two PCs with the explained variance.

Flavonoid-targeted metabolomics analysis revealed that PCA identified PC1 (31.60%) and PC2 (26.00%) as the two main components in eight studied soybean varieties (Figure 4). Thirty-five flavonoids were analyzed in soybean seeds. Only 12 flavonoids, puerarin, glycitein, daidzin, glycitin, daidzein, genistein/apigenin, isoquercitrin/hyperoside, taxifolin, luteolin, and p-coumaric acid, were detected and quantified (Table S11). The total amount of flavonoids in three natural genotypic soybean varieties ranged from 305.07 to 381.08 μg/g. The content of flavonoids in five herbicide-tolerant soybean varieties was 327.02 to 665.93 μg/g. Except for ZLD2426, the flavonoid content in the other four herbicide-tolerant soybean varieties is significantly higher than that in the three natural genotypic soybean varieties (Table 5).

**Figure 5:**
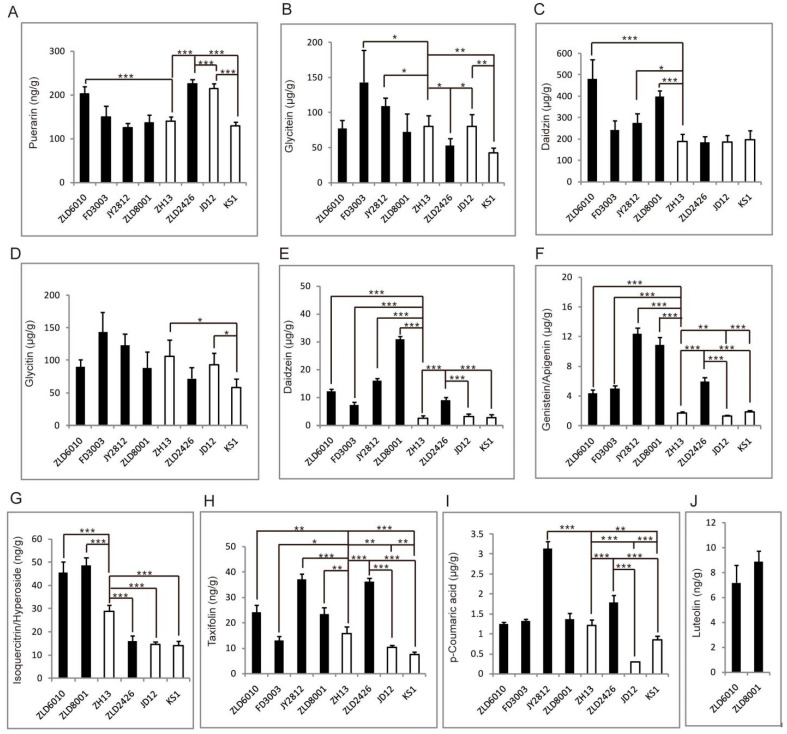
The identified flavonoid abundance pattern analysis. Puerarin (**A**), glycitein (**B**), daidzin (**C**), glycitin (**D**), daidzein (**E**), genistein/apigenin (**F**), isoquercitrin/hyperoside (**G**), taxifolin (**H**), *p*-coumaric acid (**I**), and luteolin (**J**). The asterisks represent significant differences as indicated by *t*-tests (* *p* < 0.05; ** *p* < 0.01 and *** *p* < 0.001).

Among the four herbicide-tolerant soybean varieties, ZLD6010, FD3003, JY2812, and ZLD8001, only the puerarin content in the ZLD6010 seeds was significantly greater than that in the ZH13 seeds. The content of puerarin in herbicide-tolerant soybean ZLD2426 seeds was significantly greater than that in seeds of three natural genotypic varieties of soybean, JD12, ZH13, and KS1. The puerarin content in JD12 seeds was the highest among the seeds of the three natural genotypic soybean varieties and was significantly greater than that in KS1 seeds (Figure 5A).

Among the herbicide-tolerant soybean varieties ZLD6010, FD3003, JY2812, and ZLD8001, the glycitein content in the FD3003 and JY2812 seeds was significantly greater than that in the ZH13 seeds. The content of glycitein in herbicide-tolerant soybean ZLD2426 seeds was significantly lower than that in seeds of two natural genotypic soybean varieties, JD12 and ZH13. The glycitein content in the KS1 seeds was also significantly lower than that in the JD12 and ZH13 seeds (Figure 5B).

Among the four herbicide-tolerant soybean varieties, ZLD6010, FD3003, JY2812, and ZLD8001, the daidzin content in the ZLD6010, JY2812, and ZLD8001 seeds was significantly greater than that in the ZH13 seeds. The content of daidzin in the ZLD2426 seeds was not significantly different from that in the seeds of three natural genotypic soybean varieties, JD12, ZH13, and KS1. There was also no significant difference in the content of daidzin among the seeds of three natural genotypic soybean varieties, JD12, ZH13, and KS1 (Figure 5C).

The glycitin content in the seeds of four herbicide-tolerant soybean varieties, ZLD6010, FD3003, JY2812, and ZLD2426, did not significantly differ from that in the seeds of ZH13. The content of glycitin in herbicide-tolerant soybean ZLD2426 seeds was not significantly different from that in seeds of three natural genotypic soybean varieties, JD12, ZH13, and KS1. In three natural genotypic varieties of soybeans, the glycitin content in JD12 and ZH13 was significantly greater than that in KS1 seeds (Figure 5D).

The daidzein content in four herbicide-tolerant soybean ZLD6010, FD3003, JY2812, and ZLD2426 seeds significantly differed from that in ZH13 seeds. The content of daidzein in herbicide-tolerant soybean ZLD2426 seeds was significantly greater than that in the seeds of the three natural genotypic soybean varieties. There was also no significant difference in the content of daidzein among the seeds of three natural genotypic soybean varieties, JD12, ZH13, and KS1 (Figure 5E).

The genistein/apigenin contents in four herbicide-tolerant soybean varieties, ZLD6010, FD3003, JY2812, and ZLD2426, were significantly greater than those in ZH13 seeds. The contents of genistein/apigenin in herbicide-tolerant soybean ZLD2426 seeds were significantly greater than those in the seeds of the three natural genotypic soybean varieties. The contents of genistein/apigenin in JD12 seeds were significantly lower than those in ZH13 and KS1 seeds (Figure 5F).

Isoquercitrin/hyperoside was not detected in herbicide-tolerant soybean FD3003 and JY2812 seeds. The isoquercitrin/hyperoside contents in ZLD6010, ZLD8001 and ZLD2426 were significantly different from those in ZH13 seeds. The contents of isoquercitrin/hyperoside in ZH13 were significantly greater than those in the seeds of the other two natural genotypic soybean varieties, JD12 and KS1 (Figure 5G).

The taxifolin content in four herbicide-tolerant soybean varieties, ZLD6010, FD3003, JY2812, and ZLD2426, significantly differed from that in ZH13 seeds. The content of taxifolin in the herbicide-tolerant soybean variety ZLD2426 was significantly greater than that in the seeds of the three natural genotypic soybean varieties JD12, ZH13, and KS1. The content of taxifolin in the seeds of three natural genotypic soybean varieties, JD12, ZH13, and KS1, significantly differed according to pairwise reciprocal comparisons (Figure 5H).

Among the four herbicide-tolerant soybean varieties (ZLD6010, FD3003, JY2812, and ZLD2426), only the p-coumaric acid content in JY2812 was significantly greater than that in ZH13. The p-coumaric acid content in herbicide-tolerant soybean ZLD2426 seeds was significantly greater than that in seeds of three natural soybean genotypes, JD12, ZH13, and KS1. The p-coumaric acid content in the seeds of three natural soybean genotypes, JD12, ZH13, and KS1, significantly differed according to pairwise reciprocal comparisons (Figure 5I).

Luteolin was detected only in herbicide-tolerant ZLD6010 and ZLD8001 soybean seeds (Figure 5J).

## 3. Discussion

Previous studies on the unintended effects of GM crops have examined either non-edible tissues that play important roles in crop growth [21,22,23,24] or the influence of environmental factors during growth [25,26,27,28]. The unintended effects of herbicide-tolerant soybean seeds, which are rich in protein and flavonoids, urgently require systematic evaluation. In this study, TMT quantitative proteomic and flavonoid-targeted metabolomics analyses were performed on seeds of five herbicide-tolerant soybean varieties, ZLD6010, FD3003, JY2812, ZLD8001, and ZLD2426, and three natural genotypic soybean varieties, JD12, ZH13, and KS1. ZLD6010, ZLD8001, and ZLD2426 carry the same exogenous genes (*gat* and *g2-epsps*). FD3003 carries the exogenous genes *pat* and *cp4-epsps*. JY2812 carries the exogenous gene *g10-epsps*. The experiment was designed in this way to allow us to evaluate the unintended effects and mine the data in many aspects.

A total of 65, 29, 56, 38, and 26 DEPs were detected in the five herbicide-tolerant soybean varieties, ZLD6010, FD3003, JY2812, ZLD8001, and ZLD2426, compared with ZH13, respectively. A total of 24 and 16 DEPs were found in the ZLD2426/JD12 and ZLD2426/KS1 comparisons, respectively. The DEPs were involved in flavonoid biosynthesis and the biosynthesis of various plant secondary metabolites. There were 27, 20, and 25 DEPs identified in the JD12/ZH13, ZH13/KS1, and JD12/KS1 comparison groups, respectively. None of these DEPs were identified as novel toxins or allergens and only changed in abundance, consistent with previous reports [4,9,29]. The 65 DEPs identified in ZLD6010/ZH13 were the most abundant among all the comparison groups, but still represented less than 1.5% of the total proteins (4371) identified. Therefore, the protein profile of herbicide-tolerant soybean seeds has not changed dramatically.

The herbicide-tolerant proteins GAT, G2 EPSPS, PAT, CP4 EPSPS, and G10 EPSPS were successfully expressed in soybean seeds and quantified by ELISA. However, only CP4 EPSPS and G10 EPSPS were identified as DEPs, which might be due to proteomic methodological limitations. EPSPS is a key enzyme in the shikimic acid pathway. It catalyzes aromatic amino acid biosynthesis in microorganisms and plants [30,31,32]. However, introducing EPSPS did not affect other shikimic acid pathway enzymes in the five herbicide-resistant soybean varieties. This could have two explanations. First, the exogenous protein levels may be insufficient to cause significant pathway-wide changes. Alternatively, the proteomic methodology may have limitations that prevent the detection of subtle changes.

The biosynthesis of flavonoids initiates with phenylalanine, a metabolic product of the shikimate pathway. EPSPS is a key regulatory enzyme in this pathway. The introduction of exogenous EPSPS (CP4 EPSPS, G10 EPSPS, and G2 EPSPS) might influence flavonoid accumulation by altering the availability of phenylalanine. In this study, the contents of 12 quantified flavonoids varied among the different soybean varieties. Luteolin was detected only in the ZLD6010 and ZLD8001 seeds. The contents of daidzein, genistein/apigenin, taxifolin, and luteolin in the seeds of herbicide-resistant soybean varieties were significantly greater than those in the seeds of natural genotypic soybean varieties. Certainly, this conclusion requires further substantiation through additional relevant research data, and future research may also focus on a longitudinal study of the same species across multiple years. The contents of daidzein, genistein/apigenin, taxifolin, and luteolin in the seeds of three natural genotypic soybean varieties also significantly differed according to pairwise reciprocal comparisons. There was no significant difference in glycitin content between GM soybeans and non-GM soybeans, but glycitin content in JD12 and ZH13 was significantly greater than that in KS1 seeds. This suggests that genotype has some influence on changes in the flavonoid metabolite profile of crops.

## 4. Materials and Methods

### 4.1. Plant Materials

The unintended effects of transgenes on seeds were investigated in herbicide-tolerant soybean varieties ZLD6010, ZLD8001, ZLD2426, FD3003, and JY2812, as well as in natural genotypic varieties JD12, ZH13, and KS1. Detailed characteristics of the studied soybean varieties are presented in Table 6. Genetically modified and natural genotype soybean seeds were cultivated and harvested under identical field conditions during the same growing season. Fully mature soybean seeds were used as the experimental material. Gene-specific polymerase chain reaction (PCR) was employed to confirm the genetic identity of the studied soybean lines. The sequences of the primers used are provided in Table S12.

### 4.2. Protein Preparation, Trypsin Digestion and TMT Labelling

Three biological replicates of seeds of 8 different soybean varieties were used for protein profiling analysis in this study. The proteins of the studied soybean seeds were prepared via the following procedure. Soybean seeds were ground in liquid nitrogen and incubated in lysis buffer. After reduced with 10 mM DTT, the suspension was sonicated and centrifuged. The protein was precipitated, air-dried, and resuspended in 100 mM TEAB (pH 8.0) containing 8M urea. The protein samples were reduced and alkylated with 50 mM iodoacetamide (IAM). After being repeatedly precipitated, centrifuged, air-dried, and resuspended, the total protein was digested with trypsin at a ratio of 1:50 (*w*/*w*) at 37 °C for 16 h. After digestion, 100 μg of peptide from each sample was TMT-labelled with TMT reagents according to the manufacturer’s protocol.

### 4.3. LC–MS/MS Analysis

The dried peptide sample was reconstituted with a 0.1% FA aqueous solution and centrifuged at 15,000 rpm for 10 min. Then, the sample solution was analyzed by HPLC–MS. For detailed mass spectrometry analysis and experimental procedures, please refer to the Supplementary Materials.

### 4.4. Metabolite Preparation

Soybean seeds of each studied variety were ground in liquid nitrogen. Total metabolites were extracted with 70% aqueous methanol at 4 °C. Following sonication for 30 min and centrifugation at 20,000 rpm for 15 min, the extracts were filtered before UPLC–MS/MS analysis.

### 4.5. Targeted Metabonomics Analysis

Six biological replicates of the different soybean varieties were analyzed for flavonoid-targeted metabolic profile. Thirty-five flavonoid standard substances (Table S13) were used to establish the multiple reaction monitoring (MRM) methodology. The retention times and MRM fragment ions were compared using the standard method and quantified by an external standard. During detection, the samples whose concentrations were higher than the range of the calibration curve were retested by dilution for the appropriate times, and the measured data after dilution were used as the quantitative results.

### 4.6. Data Analysis

Protein identification was performed against the UniProt soybean database supplemented with the foreign proteins GAT, G2 EPSPS, PAT, CP4 EPSPS, and G10 EPSPS. All identified proteins were matched with at least one unique peptide at ≥95% confidence [33,34]. Proteins were considered differentially expressed proteins (DEPs) based on a fold change ≥ 2.0 or ≤0.50 and *p* ≤ 0.05 [18,35,36] in the comparison groups (Table 7). A heatmap based on the hierarchical cluster analysis method was generated by Genesis software (version 2.1.6). Principal component analysis (PCA) was performed with R software (version 4.3.1, www.r-project.org). Kyoto Encyclopedia of Genes and Genomes (KEGG) pathway enrichment analysis of the DEPs was carried out using the KEGG database (http://www.genome.jp/kegg/) [37,38]. The calibration curves of each flavonoid are listed in Table S1. Significant differences in the levels of each flavonoid among the seeds of different soybean varieties were indicated by *t*-tests (* *p* < 0.05, ** *p* < 0.01, and *** *p* < 0.001).

### 4.7. ELISA (Enzyme-Linked Immunosorbent Assay) of the Foreign Proteins

Total proteins were extracted with lysis buffer using an ELISA kit. The contents of the foreign proteins G2 EPSPS, GAT, CP4 EPSPS, PAT, and G10 EPSPS were measured using ELISA kits according to the manufacturer’s instructions (Youlong, Shanghai, China, and Biotechnology Research Institute, CAAS, Beijing, China).

## 5. Conclusions

The proteomic profile of the herbicide-tolerant soybean variety examined did not exhibit significant alterations compared to that of the natural genotypic varieties. The introduction of the EPSPS into the herbicide-tolerant soybean lines might influence flavonoid accumulation relative to the natural genotypic varieties. Furthermore, the genetic background of soybean appears to exert a measurable effect on its flavonoid metabolomic profile.

## Figures and Tables

**Table 1:** Summary of the number and regulatory state of the DEPs in the different comparison groups.

Comparison Groups	No. of Upregulated Proteins	No. of Downregulated Proteins	No. of DEPs
ZLD6010/ZH13	29	36	65
FD3003/ZH13	15	14	29
JY2812/ZH13	21	35	56
ZLD8001/ZH13	18	20	38
ZLD2426/ZH13	8	18	26
ZLD2426/JD12	9	15	24
ZLD2426/KS1	6	10	16
JD12/ZH13	13	14	27
ZH13/KS1	12	8	20
JD12/KS1	15	10	25

**Table 2:** DEPs among the five GM soybean varieties compared with ZH13.

Accession	Name	Regulatory State		
		ZLD6010/ZH13 JY2812/ZH13	FD3003/ZH13	ZLD8001/ZH13 ZLD2426/ZH13
I1MAE7	Acyl-[acyl-carrier-protein] desaturase	Down	Down	Down
C6SYE0	40S ribosomal protein S21	Down	Down	Down
A0A0R4J4R5	Uncharacterized protein	Up	Up	Up
A0A0R0JI98	Translation elongation factor EF1B beta/delta subunit guanine nucleotide exchange domain-containing	Down	Up	Down

**Table 3:** DEPs among ZLD2426 compared with ZH13, JD12, and KS1.

Accession	Name	Regulation State		
		ZLD2426/ZH13	ZLD2426/JD12	ZLD2426/KS1
A0A0R4J2M7	40S ribosomal protein S4	Down	Down	Down
I1KQ93	phosphoglucomutase	Down	Down	Down
P24337	Hydrophobic seed protein	Down	Down	Down

**Table 4:** Exogenous proteins detected by proteomics and ELISA.

Comparison Group	Foreign Proteins	Fold Change	*p*-Value	Foreign Protein Content in GM Seeds (ng/g)
ZLD6010/ZH13	G2 EPSPS	-	-	79.25
	GAT	-	-	14.78
ZLD8001/ZH13	G2 EPSPS	-	-	150.84
	GAT	-	-	34.71
ZLD2426/ZH13	G2 EPSPS	-	-	93.66
	GAT	-	-	71.43
FD3003/ZH13	CP4 EPSPS	18.47	0.000048	186.05 (μg/g)
	PAT	-	-	4.36 (μg/g)
JY2812/ZH13	G10 EPSPS	15.05	0.000018	19.80 (μg/g)

-, values are not within the specified range (FC ≥ 2.0 or ≤0.50, *p* ≤ 0.05).

**Table 5:** The content of flavonoids in the studied soybean variety seeds.

Soybean Varieties	Flavonoids Content (μg/g)	Significant Difference (Compared to)		
		JD12	ZH13	KS1
ZLD6010	665.93 ± 102.24	**	**	***
FD3003	542.12 ± 99.55	*	*	**
JY2812	538.77 ± 64.27	**	**	***
ZLD8001	601.57 ± 70.95	**	**	***
ZLD2426	327.02 ± 42.13	-	-	-
JD12	363.90 ± 58.32			
ZH13	381.08 ± 53.44			
KS1	305.07 ± 51.46			

*, *p* < 0.05, **, *p*< 0.01, ***, *p* < 0.001, -, no significant difference.

**Table 6:** Summary of the studied soybean varieties.

	Soybean Varieties	Foreign Proteins
Natural genotypic varieties	JD12	no
	ZH13	
	KS1	
Herbicide-tolerant varieties	ZLD6010	G2 EPSPS GAT
	ZLD8001	
	ZLD2426	
	FD3003	CP4 EPSPS PAT
	JY2812	G10 EPSPS

**Table 7:** Summary of the comparison groups.

Comparison Type	Comparison Groups
Genetically modified compared with the natural genotype	ZLD6010/ZH13
	FD3003/ZH13
	JY2812/ZH13
	ZLD8001/ZH13
	ZLD2426/ZH13
	ZLD2426/JD12
	ZLD2426/KS1
Pairwise comparisons among natural genotypes	JD12/ZH13
	ZH13/KS1
	JD12/KS1

## Data Availability

The original contributions presented in this study are included in the article/Supplementary Material. Further inquiries can be directed to the corresponding authors.

## Supplementary Materials

The following supporting information can be downloaded at: https://www.mdpi.com/article/10.3390/ijms27020734/s1. References [39,40,41] are cited in Supplementary Materials.

## Abbreviations

The following abbreviations are used in this manuscript:

TMT	Tandem mass tag
FC	Fold change
DEPs	Differentially expressed proteins
co-DEPs	Commonly differentially expressed proteins
KEGG	Kyoto Encyclopedia of Genes and Genomes
MS	Mass spectrometry
ELISA	Enzyme-linked immunosorbent assay

## References

[1] Nichols V., Verhulst N., Cox R., Govaerts B. (2015). Weed dynamics and conservation agriculture principles. Field Crops Res..

[2] http://www.isaaa.org/gmapprovaldatabase/default.asp.

[3] Nandula V.K. (2019). Herbicide resistance traits in maize and soybean: Current status and future outlook. Plants.

[4] Ren Y., Lv J., Wang H., Li L., Peng Y., Qu L.J. (2009). A comparative proteomics approach to detect unintended effects in transgenic Arabidopsis. J. Genet. Genom. Yi Chuan Xue Bao.

[5] Cellini F., Chesson A., Colquhoun I., Constable A., Davies H.V., Engel K.H., Gatehouse A.M., Karenlampi S., Kok E.J., Leguay J.J. (2004). Unintended effects and their detection in genetically modified crops. Food Chem. Toxicol..

[6] Li X., He X.Y., Luo Y.B., Xiao G.Y., Jiang X.B., Huang K.L. (2008). Comparative analysis of nutritional composition between herbicide-tolerant rice with bar gene and its non-transgenic counterpart. J. Food Compos. Anal..

[7] Han J.H., Yang Y.X., Chen S.R., Wang Z., Yang X.L., Wang G.D., Men J.H. (2005). Comparison of nutrient composition of parental rice and rice genetically modified with cowpea trypsin inhibitor in China. J. Food Compos. Anal..

[8] Ricroch A.E., Berge J.B., Kuntz M. (2011). Evaluation of Genetically Engineered Crops Using Transcriptomic, Proteomic, and Metabolomic Profiling Techniques. Plant Physiol..

[9] Gong C.Y., Li Q., Yu H.T., Wang Z.Z., Wang T. (2012). Proteomics Insight into the Biological Safety of Transgenic Modification of Rice As Compared with Conventional Genetic Breeding and Spontaneous Genotypic Variation. J. Proteome Res..

[10] Kuiper H.A., Kok E.J., Engel K.H. (2003). Exploitation of molecular profiling techniques for GM food safety assessment. Curr. Opin. Biotech..

[11] Baudo M.M., Lyons R., Powers S., Pastori G.M., Edwards K.J., Holdsworth M.J., Shewry P.R. (2006). Transgenesis has less impact on the transcriptome of wheat grain than conventional breeding. Plant Biotechnol. J..

[12] Barros E., Lezar S., Anttonen M.J., van Dijk J.P., Rohlig R.M., Kok E.J., Engel K.H. (2010). Comparison of two GM maize varieties with a near-isogenic non-GM variety using transcriptomics, proteomics and metabolomics. Plant Biotechnol. J..

[13] Coll A., Nadal A., Rossignol M., Puigdomenech P., Pla M. (2011). Proteomic analysis of MON810 and comparable non-GM maize varieties grown in agricultural fields. Transgenic Res..

[14] Scossa F., Laudencia-Chingcuanco D., Anderson O.D., Vensel W.H., Lafiandra D., D’Ovidio R., Masci S. (2008). Comparative proteomic and transcriptional profiling of a bread wheat cultivar and its derived transgenic line overexpressing a low molecular weight glutenin subunit gene in the endosperm. Proteomics.

[15] Kok E.J., Kuiper H.A. (2003). Comparative safety assessment for biotech crops. Trends Biotechnol..

[16] Chassy B.N. (2002). Food safety evaluation of crops produced through biotechnology. J. Am. Coll. Nutr..

[17] Brandao A.R., Barbosa H.S., Arruda M.A.Z. (2010). Image analysis of two-dimensional gel electrophoresis for comparative proteomics of transgenic and non-transgenic soybean seeds. J. Proteom..

[18] Wang L.M., Wang X.C., Jin X., Jia R.Z., Huang Q.X., Tan Y.H., Guo A.P. (2015). Comparative proteomics of Bt-transgenic and non-transgenic cotton leaves. Proteome Sci..

[19] Wang Y., Xu W.T., Zhao W.W., Hao J.R., Luo Y.B., Tang X.G., Zhang Y., Huang K.L. (2012). Comparative analysis of the proteomic and nutritional composition of transgenic rice seeds with Cry1ab/ac genes and their non-transgenic counterparts. J. Cereal Sci..

[20] Khalf M., Goulet C., Vorster J., Brunelle F., Anguenot R., Fliss I., Michaud D. (2010). Tubers from potato lines expressing a tomato Kunitz protease inhibitor are substantially equivalent to parental and transgenic controls. Plant Biotechnol. J..

[21] Plischke A., Choi Y.H., Brakefield P.M., Klinkhamer P.G.L., Bruinsma M. (2012). Metabolomic Plasticity in GM and Non-GM Potato Leaves in Response to Aphid Herbivory and Virus Infection. J. Agric. Food Chem..

[22] Zhou J., Zhang L., Chang Y.W., Lu X., Zhu Z., Xu G.W. (2012). Alteration of Leaf Metabolism in Bt-Transgenic Rice (*Oryza sativa* L.) and Its Wild Type under Insecticide Stress. J. Proteome Res..

[23] Christ B., Hochstrasser R., Guyer L., Francisco R., Aubry S., Hortensteiner S., Weng J.K. (2017). Non-specific activities of the major herbicide-resistance gene BAR. Nat. Plants.

[24] Hao W.Y., Li F.W., Yan W., Li C.C., Hao D.Y. (2017). Comparative metabolic profiling of four transgenic maize lines and two non-transgenic maize lines using high-performance liquid chromatography mass spectrometry. Acta Physiol. Plant.

[25] Chang Y.W., Zhao C.X., Zhu Z., Wu Z.M., Zhou J., Zhao Y.N., Lu X., Xu G.W. (2012). Metabolic profiling based on LC/MS to evaluate unintended effects of transgenic rice with cry1Ac and sck genes. Plant Mol. Biol..

[26] Frank T., Rohlig R.M., Davies H.V., Barros E., Engel K.H. (2012). Metabolite Profiling of Maize Kernels-Genetic Modification versus Environmental Influence. J. Agric. Food Chem..

[27] Chen M.J., Rao R.S.P., Zhang Y.M., Zhong C., Thelen J.J. (2016). Metabolite variation in hybrid corn grain from a large-scale multisite study. Crop J..

[28] Tang W.J., Hazebroek J., Zhong C., Harp T., Vlahakis C., Baumhover B., Asiago V. (2017). Effect of Genetics, Environment, and Phenotype on the Metabolome of Maize Hybrids Using GC/MS and LC/MS. J. Agric. Food Chem..

[29] Arruda S.C., Barbosa H.S., Azevedo R.A., Arruda M.A. (2013). Comparative studies focusing on transgenic through cp4EPSPS gene and non-transgenic soybean plants: An analysis of protein species and enzymes. J. Proteom..

[30] Zabalza A., Orcaray L., Fernandez-Escalada M., Zulet-Gonzalez A., Royuela M. (2017). The pattern of shikimate pathway and phenylpropanoids after inhibition by glyphosate or quinate feeding in pea roots. Pestic. Biochem. Phys..

[31] Herrmann K.M. (1995). The Shikimate Pathway-Early Steps in the Biosynthesis of Aromatic-Compounds. Plant Cell.

[32] Mir R., Jallu S., Singh T.P. (2015). The shikimate pathway: Review of amino acid sequence, function and three-dimensional structures of the enzymes. Crit. Rev. Microbiol..

[33] Qin J., Zhang J.N., Wang F.M., Wang J.H., Zheng Z., Yin C.C., Chen H., Shi A.N., Zhang B., Chen P.Y. (2017). iTRAQ protein profile analysis of developmental dynamics in soybean [*Glycine max* (L.) Merr.] leaves. PLoS ONE.

[34] Baldrianova J., Cerny M., Novak J., Jedelsky P.L., Diviskova E., Brzobohaty B. (2015). Arabidopsis proteome responses to the smoke-derived growth regulator karrikin. J. Proteom..

[35] Zeng W.Y., Sun Z.D., Cai Z.Y., Chen H.Z., Lai Z.G., Yang S.Z., Tang X.M. (2017). Proteomic analysis by iTRAQ-MRM of soybean resistance to Lamprosema Indicate. BMC Genom..

[36] Liu Y.B., Zhang Y.X., Song S.Q., Li J.S., Stewart C.N., Wei W., Zhao Y.J., Wang W.Q. (2015). A proteomic analysis of seeds from Bt-transgenic Brassica napus and hybrids with wild B. juncea. Sci. Rep..

[37] Kanehisa M., Goto S. (2000). KEGG: Kyoto encyclopedia of genes and genomes. Nucleic Acids Res..

[38] Ogata H., Goto S., Sato K., Fujibuchi W., Bono H., Kanehisa M. (1999). KEGG: Kyoto Encyclopedia of Genes and Genomes. Nucleic Acids Res..

[39] Huang D.W., Sherman B.T., Lempicki R.A. (2009). Bioinformatics enrichment tools: Paths toward the comprehensive functional analysis of large gene lists. Nucleic Acids Res..

[40] Sun Z., Li L., Qu J., Li H., Chen H. (2019). Proteomic analysis of therapeutic effects of Qingyi pellet on rodent severe acute pancreatitis-associated lung injury. Biomed. Pharmacother..

[41] Livak K.J., Schmittgen T.D. (2001). Analysis of relative gene expression data using real-time quantitative PCR and the 2^−ΔΔCT^ method. Methods.

