# Supplementary material for: Evaluation of the Unintended Effects of Herbicide-Resistant Soybean Seeds via TMT Quantitative Proteomics and Flavonoid-Targeted Metabolomics
Source: Int J Mol Sci. 2026 Jan 11;27(2):734. doi: 10.3390/ijms27020734 (PMC12840600; doi:10.3390/ijms27020734)
Supplement: Supplementary file 1 [file ijms-27-00734-s001.zip › Sup 2revised.pdf]

Article

Evaluation of the Unintended Effects of Herbicide-Resistant Soybean Seeds by TMT Quantitative Proteomics and Flavonoid-targeted Metabolomics

Zhanchao Wang [1], Ruizhe Wang [1], Mei Dong [1], Guihua Hu [1], Chaohua Miao [1], Yusong Wan [1], Weixiao Liu [1,*], and Wujun Jin [1, 2,*]

[1] Biotechnology Research Institute, Chinese Academy of Agricultural Sciences, Beijing 100081, China; w1551725922@126.com (Z.W.); 2218763231@qq.com (R.W.); dmei2010@126.com (M.D.); huguihua@caas.cn (G.H.); miaochaohua@caas.cn (C.M.); wanyusong@caas.cn (Y.W.)

[2] National Nanfan Research Institute, Chinese Academy of Agricultural Sciences, Sanya 572024, China

* Correspondence: jinwujun@caas.cn and liuweixiao@caas.cn

Materials and methods

1. Protein preparation, Trypsin digestion and TMT Lableing

Soybean seeds were ground in liquid nitrogen and incubated in lysis buffer (Genecreat, Wuhan, China) (7 M urea, 2 M thiourea, 4% SDS, and 40 mM Tris-HCl (pH 8.5)) containing 1 mM PMSF and 2 mM EDTA (final concentration) for 5 min, then 10 mM DTT (Sigma, Shanghai, China) was added to the sample. The suspension was sonicated for 10 min on ice and then centrifuged at 4 °C and 13,000 rpm for 20 min. The supernatant was mixed with 4 volumes of precooled acetone at −20 °C for 2 h. After centrifugation, the protein pellets were air-dried and resuspended in 8 M urea/100 mM TEAB (Sigma, Shanghai, China). The protein samples were reduced with 10 mM DTT at 56 °C for 30 min and then alkylated with 50 mM iodoacetamide (IAM) (Sigma, Shanghai, China) at room temperature for 30 min in the dark. Next, 4 volumes of precooled acetone were added at −20 °C for 2 h. After centrifugation, the protein pellets were air-dried and resuspended in 8 M urea(Sigma, Shanghai, China) /100 mM TEAB (pH 8.0). Then, the total protein concentration was measured using the Bradford method.

Trypsin (MS grade, Sigma, Shanghai, China)and 100 μg protein from each sample were mixed at an enzyme–protein ratio of 1:50 (w/w). Digestion was performed at 37 °C for 16 h. After digestion, the peptides were desalted using C18 columns and dried with a vacuum concentration.

Take out the TMT reagent and thaw it at room temperature. Open the cap and add

41 μl of acetonitrile. Shake for 5 minutes and centrifuge. Add the TMT reagent to 100 μg of the digested sample and react at room temperature for 1 hour. Add ammonia water to terminate the reaction. Mix the labeled samples, vortex, and centrifuge until the bottom of the tube. Vacuum freeze-dry.

2. LC–MS/MS analysis

Prepare mobile phase A (100% water, 0.1% formic acid) and B (80% acetonitrile, 0.1% formic acid). Dissolve the lyophilized powder with 10 μL of A solution, centrifuge at 14,000 g for 20 min at 4°C, and inject 1 μg of the supernatant sample for LC-MS analysis. The LC elution conditions are shown in Table 1. Use a Q Exactive HF-X mass spectrometer with a Nanospray Flex™ (NSI) ion source. Set the ion spray voltage to 2.2 kV and the ion transfer tube temperature to 320°C. The mass spectrometer operates in data-dependent acquisition mode, with a full scan range of m/z 350-1800, a resolution of 60,000 (at 200 m/z) for the first mass spectrometry, an AGC of 3×106, and a maximum injection time of 50 ms for the C-trap. Select the top 15 ions with the highest intensity from the full scan as precursor ions and fragment them using the high-energy collision dissociation (HCD) method for the second mass spectrometry. Set the resolution for the second mass spectrometry to 45,000 (at 200 m/z), an AGC of 2×105, a maximum injection time of 120 ms, and a peptide fragmentation collision energy of 34%. Generate the raw mass spectrometry data (.raw).

3. UPLC-ESI-MS/MS analysis

The extracted flavonoids were analyzed using an UPLC-ESI-MS/MS system (UPLC, Shim-pack UFLC SHIMADZU CBM30A system; MS, Applied Biosystems 4500 Q TRAP). The analytical conditions were as follows: UPLC: column, Agilent SB-C18 (1.8 μm, 2.1 mm ×100 mm); mobile phase consisting of solvent A (98% DDH_2O, 0.1% FA) and solvent B (ACN). Sample measurements were performed with a gradient program that employed the starting conditions of 95% A and 5% B. Within 9 min, a linear gradient to 5% A and 95% B was programmed, and a composition of 5% A and 95% B was maintained for 1 min. Subsequently, the composition was adjusted to 95% A and 5% B within 1.10 min and maintained for 2.90 min. The column oven was set to 40 °C, while the injection volume was 4 μL. The effluent was alternatively connected to an ESI-triple quadrupole-linear ion trap (QTRAP)-M.

The ESI source operation parameters were as follows: ion source and turbo spray; source temperature of 550 ℃; ion spray voltage (IS) of 5500 V (positive ion mode)/−4500 V (negative ion mode); ion source gas I (GSI), gas II (GSII), and curtain gas (CUR) set at 50, 60, and 30.0 psi, respectively; high collision gas (CAD). Instrument tuning and mass calibration were performed with 10 and 100 μmol/L polypropylene glycol solutions in QQQ and LIT modes, respectively. QQQ scans were acquired as MRM experiments with the collision gas (nitrogen) set to 5 psi. DP and CE for individual MRM transitions were carried out with further DP and CE optimization. A specific set of MRM transitions were monitored for each period according to the metabolites eluted within this period.

4. Data analysis

Maxquant1.6.17.0 was used to retrieve and analyze the mass spectrometry data. Protein identification was performed against the UniProt Glycine max (soybean) database supplemented with five foreign proteins, namely, GAT, G2 EPSPS, PAT, CP4 EPSPS, and G10 EPSPS. The retention time and MRM fragment ion were qualitatively compared with the standard method, and the external standard method was used for quantification.

For functional protein analysis and differential expression protein (DEP) identification, median normalization was performed on the raw data to minimize experimental variability. Subsequently, samples with more than 50% missing values were excluded from further analysis. Missing values in the remaining dataset were imputed using the Perseus algorithm. Differentially expressed proteins (DEPs) were defined based on the following criteria: a minimum average fold change of 2.0 (up or down-regulated) and a statistical significance level of $p < 0.05$.

The databases COG (Clusters of Orthologous Groups) and KEGG (Kyoto Encyclopedia of Genes and Genomes) were used to analyze the protein family and pathway [1]. GO annotation of the proteome was derived from the GO database (https://www.ebi.ac.uk/QuickGO/)[3]. Proteins were classified by GO annotation based on three categories as follows: molecular function, biological process, and cellular component. The pathway analysis was performed using the KEGG pathway (KEGG protein database: http://www.kegg.jp/kegg/pathway.html) [2]. The KEGG database was used in this study to identify enriched pathways and to test the enrichment of the different proteins against all identified proteins by Hypergeometric

distribution.

[1] Huang da, W., B.T. Sherman, and R.A. Lempicki. (2009). Bioinformatics enrichment tools: paths toward the comprehensive functional analysis of large gene lists. Nucleic Acids Res 37: 1-13.

[2] Sun Zhongwei,Li Lei,Qu Jialin et al. Proteomic analysis of therapeutic effects of Qingyi pellet on rodent severe acute pancreatitis-associated lung injury.[J] .Biomed Pharmacother, 2019, 118: 109300.

5. qRT-PCR

Approximately 1.0 g of seeds per soybean line was ground in liquid nitrogen and used for total RNA extraction with Ambion Pure Link Plant RNA Reagent (Life Technologies, Invitrogen, Carlsbad, CA, USA), following the manufacturer's instructions. RNA integrity was assessed by agarose gel electrophoresis. Subsequently, 1.0 μg of RNA was reverse transcribed using M-MLV reverse transcriptase (Life Technologies, Invitrogen) according to the manufacturer's protocol. Gene-specific primers for quantitative real-time PCR (qRT-PCR) are listed in Supplementary Table S12 and were designed using Primer Premier 5.0. qRT-PCR was carried out using the SYBR Green qRT-PCR Kit (Bio-Rad, Hercules, CA, USA) in accordance with the manufacturer’s instructions, with three biological replicates. All reactions were conducted on a CFX96 Real-Time PCR System (Bio-Rad). The qRT-PCR data were analysed

using the $2^{-\Delta\Delta CT}$ relative quantification method [3].The expression of actin was quantified as an internal control.

[3] Livak, K.J., and Schmittgen, T.D. (2001) Analysis of relative gene expression data using real-time quantitative PCR and the 2(T)(-Delta Delta C) method. Methods. 25, 402-408

Table S1 to S10 are provided as Excel files..

Table S11. The content of flavonoids detected by targeted metabolomics (Puerarin, Isoquercitrin/Hyperoside, Taxifolin andLuteolin (ng/g) and Glycitein, Daidzin, Glycitin, Daidzein, Genistein/Apigeninand p-Coumaric acid (μg/g)).

**Compound**	**ZLD6010**	**FD3003**	**JY2812**	**ZLD8001**	**ZLD2426**	**JD12**	**ZH13**	**KS1**
Puerarin	202.33±16.51	149.67±24.55	125.17±9.97	136.33±18.25	140.00±10.58	225.00±10.37	214.50±11.59	129.17±9.54
Glycitein	76.95±12.45	143.25±45.98	109.48±11.52	72.00±26.71	80.55±14.87	52.58±10.55	79.92±17.55	42.65±6.91
Daidzin	480.50±91.60	241.17±44.17	274.50±44.31	398.00±28.49	188.33±36.15	185.00±27.38	185.67±31.22	198.33±40.53
Glycitin	90.50±10.19	143.93±30.54	123.13±17.94	88.12±25.35	106.57±24.68	72.35±16.55	93.32±18.33	58.40±13.38
Daidzein	12.12±0.92	7.28±0.84	15.95±1.62	30.97±2.38	2.52±0.23	9.07±0.42	3.18±0.61	2.80±0.36
Genistein&Apigenin	4.35±0.50	5.015±0.36	12.40±0.78	10.90±0.99	1.72±0.16	5.95±0.52	1.29±0.16	1.88±0.19
Isoquercitrin& Hyperoside	45.63±4.62	-	-	48.50±3.61	28.93±2.61	15.97±2.47	14.70±1.17	14.15±1.75
Taxifolin	24.10±2.97	12.97±1.65	37.05±2.22	23.42±2.56	15.85±2.60	36.17±1.39	10.29±1.88	7.55±1.02
Luteolin	7.19±1.42	-	-	8.89±0.84	-	-	-	-
p-Coumaric acid	1.24±0.05	1.32±0.05	3.14±0.17	1.37±0.15	1.21±0.14	1.79±0.18	0.30±0.01	0.86±0.10

-, not detected.

Table S12. Primers for confirm the genetic identity of the studied soybean lines and qRT-PCR detection of coDEP.

Primer	Sequence (5'-3')
g2 epsps F	atggcgtgtt tgcctgatga ttcg
g2 epsps R	TCAGTCGTTT AGGTGAACGC CCAGG
gat F	ATGATAGAGG TGAAACCGAT TAACG
gat R	TTATGTGATC CTTTTATACA TCAG
cp4 epsps F	ATGC TTCACGGTGC AAGCAGCCGG
cp4 epsps R	TCAGGCAGCC TTCGTATCGG AGAG
pat F	ATGTCTCCGG AGAGGAGACC AGTTG
pat R	TCAGATCTGG GTAACTGGCC TAAC
g10 epsps F	ATGGCCACCTTCGACGTGATCGTG
g10 epsps R	TTAGGCGGTG GCCTCAGCGT ACTCG
actin F	CCGGTCGTGACCTCACTGATTTCT
actin R	CATCAGGCAACTCGTAGCTCTTCTCG
LOC100777627 F	GGATCTCTAGTTGCAGAGCCT
LOC100777627 R	CCCAAAGCAACCCTTCCATCT

Table S13. The information of the standard substances.

No.	The standard substances	Molecular Formula	Molecular Weight	retention time （min）	linear range （ng/mL）	linear equation	r	LOQ (ng/g）
1	Rutin	$C_{27}H_{30}O_{16}$	610.52	5.69	5.00~50.0	y = 3173.94305 x -5401.53191	0.9996	0.500
2	Diosmetin	$C_{16}H_{12}O_{6}$	300.26	8.83	0.500~10.0	y = 411.48056 x + 2292.59885	0.9992	0.500
3	Hesperetin	$C_{16}H_{14}O_{6}$	302.28	8.92	5.00~100	y = 4429.63881 x -2155.55099	0.9995	0.500
4	Nobiletin	$C_{21}H_{22}O_{8}$	402.39	10.73	10.0~100	y = 21322.44664 x + 4.50631e4	0.9993	0.250
5	Demethylnobiletin	$C_{20}H_{20}O_{8}$	388.37	12.17	5.00~100	y = 13413.75202 x -907.58521	0.9990	0.250
6	3-Methoxyflavone	$C_{16}H_{12}O_{3}$	252.26	11.95	0.500~10.0	y = 5.26052e4 x + 4501.01415	0.9950	0.125
7	5-Methoxyflavone	$C_{16}H_{12}O_{3}$	252.26	10.19	5.00~100	y = 15634.84912 x + 27943.48587	0.9996	0.250
8	6-Methoxyflavone	$C_{16}H_{12}O_{3}$	252.26	11.65	0.500~10.0	y = 4.80356e4 x + 1165.23271	0.9998	0.125
9	7-Methoxyflavone	$C_{16}H_{12}O_{3}$	252.26	11.23	2.00~50.0	y = 13267.88634 x -1548.11672	0.9995	0.250
10	3,3',4',5,6,7,8-heptamethoxyflavone	$C_{22}H_{24}O_{9}$	432.43	11.21	0.500~10.0	y = 13336.37952 x -1084.65301	0.9997	0.125
11	5,7,8,4'-tetramethoxyflavone	$C_{19}H_{18}O_{6}$	342.34	10.04	1.00~20.0	y = 3.05617e4 x + 4492.16592	0.9997	0.250
12	Tangeretin	$C_{20}H_{20}O_{7}$	372.1	11.51	0.500~10.0	y = 6.45187e5 x + 5.43343e4	0.9995	0.125
13	Naringin	$C_{27}H_{32}O_{14}$	580.53	6.40	20.0~500	y = 2002.54749 x + 8816.83722	0.9999	1.25
14	Hesperidin	$C_{28}H_{34}O_{15}$	610.2	6.55	1.00~10.0	y = 2282.34332 x -602.87332	0.9963	0.250
15	Synephrine	$C_{9}H_{13}NO_{2}$	167.21	0.74	5.00~100	y = 1.04954e5 x + 1.69716e5	0.9995	0.250
16	Quercetin	$C_{15}H_{10}O_{7}$	302.24	8.9	50.0~1000	y = 345.25766 x + 2874.85576	0.9996	2.50
17	Cynaroside	$C_{21}H_{20}O_{11}$	448.38	5.94	1.00~20.0	y = 3522.49661 x + 2151.97496	0.9991	0.250
18	Myricetin	$C_{15}H_{10}O_{8}$	318.24	6.77	5.00~50.0	y = 12977.12041 x -67308.0	0.9987	1.25
19	Puerarin	$C_{21}H_{20}O_{10}$	432.38	4.7	1.00~20.0	y = 4295.24299 x -270.52891	0.9995	0.500

20	Glycitein	$C_{16}H_{12}O_5$	284.26	5.38	20.0~500	y = 1410.55512 x -8721.29683	0.9999	1.25
21	Daidzin	$C_{21}H_{20}O_9$	416.38	5.25	100~1000	y = 180.53116 x + 4023.15166	0.9973	2.50
22	Glycitin	$C_{22}H_{22}O_{10}$	446.4	5.37	20.0~200	y = 1305.63474 x + 22882.77462	0.9968	1.25
23	Daidzein	$C_{15}H_{10}O_4$	254.24	7.41	2.00~50.0	y = 755.91349 x -627.22867	0.9993	0.500
24	Dihydromyricetin	$C_{15}H_{12}O_8$	320.25	4.94	5.00~100	y = 5249.88927 x-6272.81724	0.9999	0.500
25	Genistein/Apigenin	$C_{15}H_{10}O_5$/ $C_{15}H_{10}O_5$	270.24/270.2 4	8.56	2.00~40.0	y =4810.94138x-538.25792	0.9987	0.500
26	Coumarin	$C_9H_6O_2$	146.14	7.11	20.0~500	y = 880.64201 x + 4293.11150	0.9998	5.00
27	Neohesperidin	$C_{28}H_{34}O_{15}$	610.56	6.72	2.00~50.0	y = 6490.86288 x -786.41083	0.9996	0.500
28	Isoquercitrin/Hyperoside	$C_{21}H_{20}O_{12}$/ $C_{21}H_{20}O_{12}$	464.1/464.38	5.81	2.00~40.0	y = 2867.48744 x -2425.84857	0.9995	0.500
29	Taxifolin	$C_{15}H_{12}O_7$	304.25	6.01	1.00~20.0	y = 10876.52617 x -332.24287	0.9991	0.250
30	Quercitrin	$C_{21}H_{20}O_{11}$	448.38	6.37	20.0~200	y = 1275.94493 x -10730.15454	0.9993	2.50
31	Luteolin	$C_{15}H_{10}O_6$	286.24	7.73	1.00~20.0	y = 18689.83584 x -4640.47712	0.9984	0.250
32	p-Coumaric acid	$C_9H_8O_3$	164.16	5.42	2.00~50.0	y = 19419.22241 x -7307.95411	0.9994	1.25
33	3-Hydroxyflavone	$C_{15}H_{10}O_3$	238.24	4.71	10.0~100	y = 713.13169 x + 4116.69686	0.9992	1.25

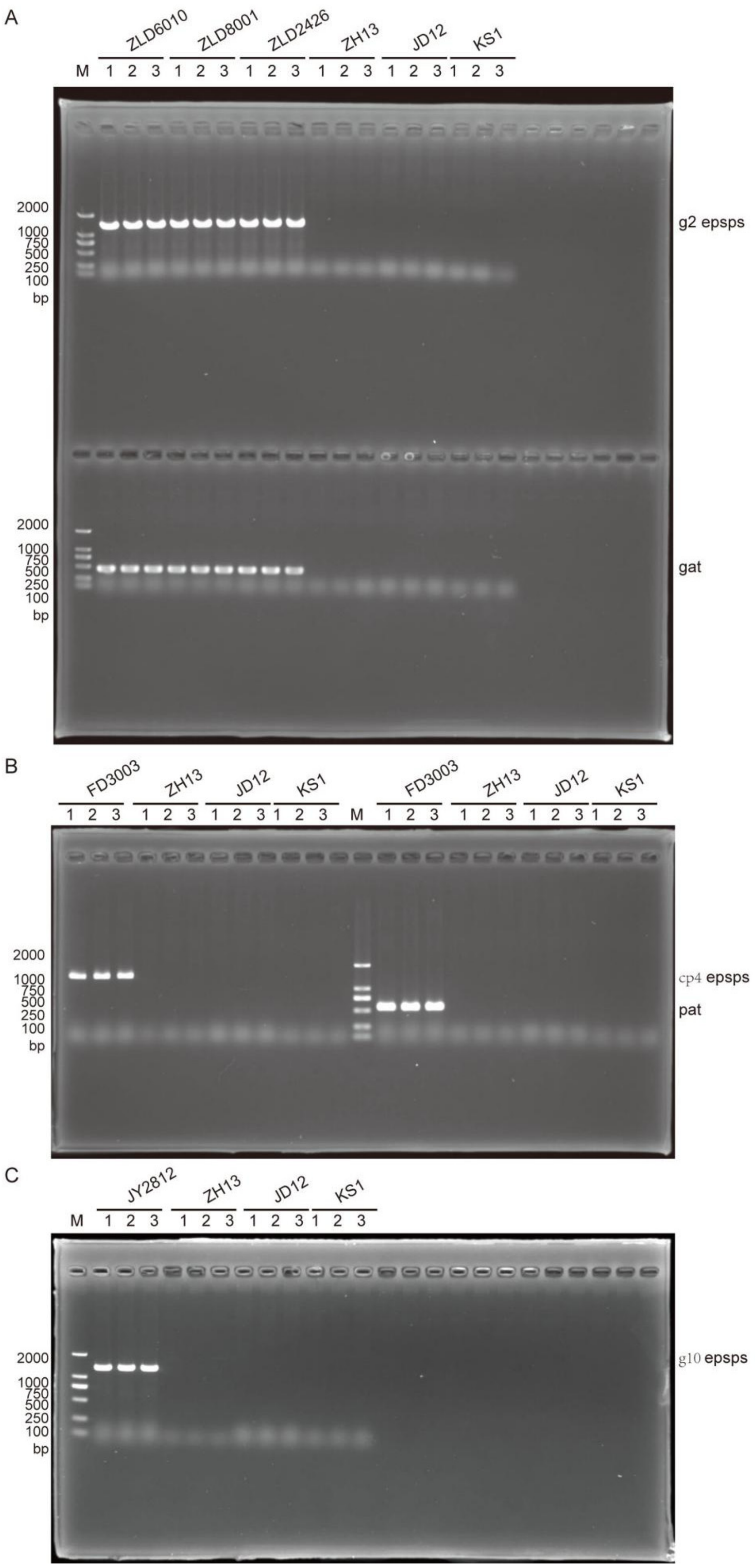

Figure S1. Gene-specific PCR-based detection of the studied soybean lines (A, ZLD6010, ZLD8001 and ZLD2426; B, FD3003; C, JY2812). M, Trans2k.

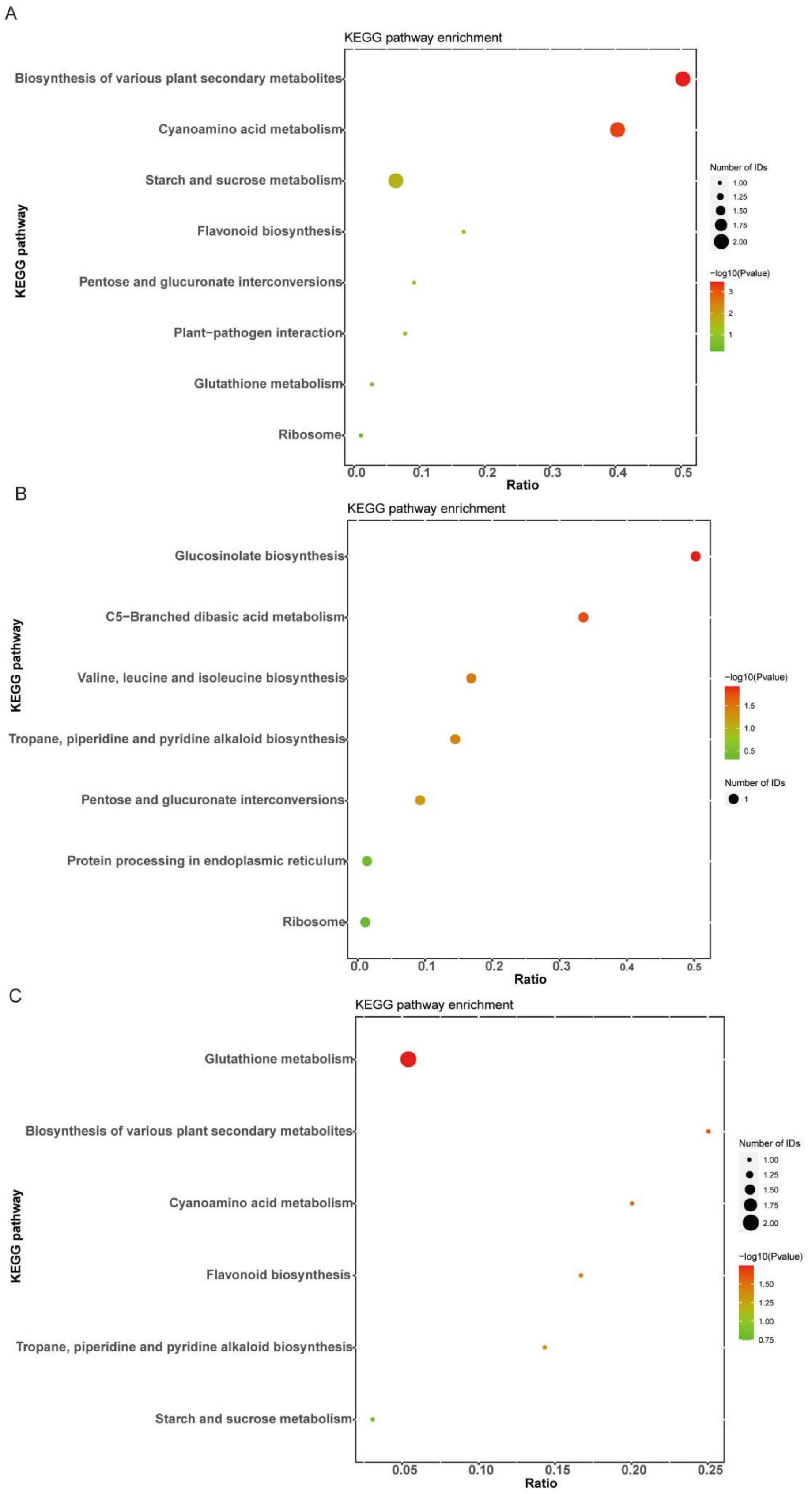

Figure S2. KEGG pathway enrichment analysis of DEPs in JD12/ZH13 (A), ZH13/KS1 (B), JD12/ KS1 (C)

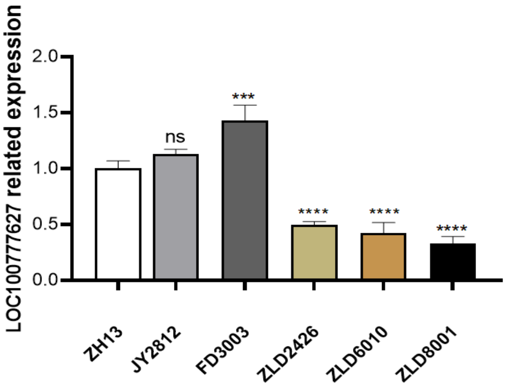

Figure S3. qRT-PCR analysis of gene expression patterns of selected coDEP (the translation elongation factor EF1B: LOC100777627).
